# Comparative study of suction drainage placement in cementless hip replacement among patients undergoing extended thromboprophylaxis: a prospective randomized study

**DOI:** 10.1186/s12891-021-04583-0

**Published:** 2021-08-13

**Authors:** Paweł Bartosz, Wojciech Marczyński, Marcin Para, Maciej Kogut, Jerzy Białecki

**Affiliations:** Orthopedic Department, Centre of Postgraduate Medical Education, Konarskiego 13, Kosciuszki 3/10B, 05-400 Otwock, Poland

**Keywords:** Suction drainage, Cementless hip replacement, Surgical outcomes, Hematoma

## Abstract

**Background:**

The use of drains reportedly does not improve surgical outcomes after hip replacement. There is still a lack of strict recommendations for drain placement after primary hip replacement. This study aimed to assess the safety of not using suction drainage after primary hip replacement in a population of patients undergoing extended thromboprophylaxis.

**Methods:**

In this prospective randomized study, all patients were qualified for primary hip replacement and were divided into two groups: with and without drainage. The inclusion criterion was idiopathic hip osteoarthritis. The exclusion criteria were secondary coxarthrosis, autoimmune disease, coagulopathy, venous/arterial thrombosis, hepatic/renal insufficiency, cement, or hybrid endoprostheses. We performed an intention-to-treat analysis. Clinical, laboratory, and radiographic parameters were measured for the first three days after surgery. Hematoma collection, due to extended thromboprophylaxis, in the joint and soft tissues was evaluated precisely. The patients underwent follow-up for 30 days.

**Results:**

The final analysis included a total of 100 patients. We did not find any significant statistical differences between groups in terms of hip fluid collection (9.76 vs. 10.33 mm, with and without drainage, respectively; mean difference, 0.6 mm; 95 % confidence interval [CI] -2.8 to 3.9; *p* = 0.653), estimated blood loss (1126 vs. 1224 ml; mean difference, 97.1 ml; 95 % CI -84.1 to 278.2; *p* = 0.59), and hemoglobin levels on postoperative day 3 (11.05 vs. 10.85 g/dl; mean difference, 0.2; 95 % CI -2.1 to 2.5; *p* = 0.53). In addition, the other parameters did not show significant differences between groups. Notably, two cases of early infections were observed in the no-drainage group, whereas there were no such complications in the drainage group.

**Conclusions:**

We conclude that the use of closed suction drainage after primary hip replacement is a safe procedure in patients undergoing extended thromboprophylaxis. Further research is warranted to validate these findings.

**Trial registration:**

The study was successfully registered retrospectively at Clinicaltrial.gov with the identification number NCT04333264 03 April 2020.

## Background

Hip replacement surgery is one of the most effective procedures developed in the last century [1]. It is an orthopedic procedure that entails soft tissue and bone damage. Intraoperative blood loss can be limited by hemostasis at several stages and by using anatomical and non-aggressive surgical techniques. The use of fibrinolysis inhibitors such as tranexamic acid, as well as the topical use of vasoconstrictors, reduces both intra- and postoperative bleeding [2].

Significant intraoperative blood loss can affect hemostasis, leading to increased postoperative bleeding [3]. Suction drainage is used to drain blood from the hip joint. It is also used to assess bleeding activity, which may be an indication for urgent surgical intervention and repeated hemostasis. Excessive hematoma formation may also serve as a medium for bacterial growth. In this situation, not using suction drainage could lead to a larger hematoma in the joint and increase the risk of infection. Unfortunately, the drainage tube is also a significant portal of entry for infections in the hip. Several studies have documented the risk of infection in the context of prolonged suction drainage [4, 5]. These factors encouraged us to perform an analysis to assess the risks of hematoma formation and infection among patients after primary hip replacement according to drainage use.

Postoperative blood loss is difficult to assess. The basic diagnostic method used was a physical examination supported by diagnostic imaging. Ultrasonography is the method of choice for assessing fluid collection in soft tissues [6].

According to the literature, proper use of thromboembolic prophylaxis, including low-molecular-weight heparin (LMWH), may reduce the rate of postoperative hematomas [7]. In Poland, it is recommended that LMWH should be administered 12 h preoperatively and continued for 35 days after the intervention, which can be considered a form of extended thromboprophylaxis [8].

Until recently, strict recommendations for suction drainage after primary total hip replacement have not been available. Our study aimed to determine whether using a drain following hip replacement surgery results in similarities in joint hematoma formation, hemoglobin and C-reactive protein (CRP) levels, Visual Analog Scale (VAS), hip range of movement, wound exudation, soft tissue hematoma formation, perioperative bleeding, and blood transfusion administration when compared to not using a drain in patients undergoing extended perioperative thromboprophylaxis.

## Methods

### Study design

This study was planned and conducted using a prospective randomized design. We performed simple randomization using closed envelopes designating group allocation with an allocation ratio of 1:1. To detect our primary outcomes sample size of 50 patients per group will be sufficient to achieve a 5 % significance level and a power of 80 % according to Fagotti’s study where VAS after surgery was 2±1.3 for the no-drainage group and 1±0.2 for the drainage group [9]. Information regarding drainage usage was enclosed in envelopes that were drawn by a person blinded to the study and randomized among the patients. Neither the patients nor the clinicians knew what procedures would be performed. The envelopes were opened in the operating theater at the end of surgery by the anesthesia team, just before the decision to leave suction drainage. If the surgeon, considering the course of the procedure and local conditions, decided that suction drainage was necessary despite randomization, the patient was excluded from the study and the envelope was not opened.

After recruitment, we evaluated the differences between the investigated groups in terms of age, sex, body weight, and blood clotting parameters to verify the randomization process. The study protocol was successfully retrospectively registered at Clinicaltrial.gov with the identification number NCT04333264 (03/04/2020).

### Participants and recruitment

The inclusion criteria were primary hip osteoarthritis and age between 30 and 80 years. The exclusion criteria were secondary degenerative hip joint disease, autoimmune disease, congenital/secondary coagulopathy, history of venous/arterial thrombosis, hepatic/renal insufficiency, cement or hybrid endoprosthesis, and lack of patient consent.

**Fig. 1:**
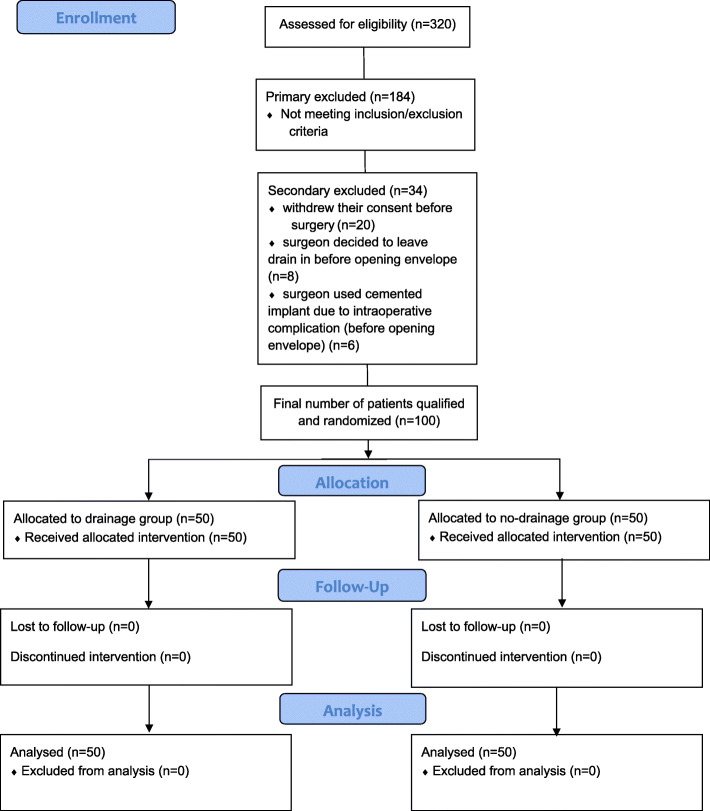
Flowchart of patient allocation and randomization process

Overall, 320 patients were successively operated on from March 14, 2016 to May 16, 2018, at the Orthopedic Department, Centre of Postgraduate Medical Education, Otwock, Poland. Only 134 patients met the inclusion criteria. All qualified patients signed written informed consent. The patients were allocated to two groups depending on the presence or absence of drains (Fig. 1); 100 patients qualified for the final analysis. Twenty patients withdrew their consent before the operation; 14 patients were excluded from the study due to intraoperative conditions (in 8 patients, the surgeon decided to leave drainage despite randomization; in 6 patients, a cemented implant was needed due to intraoperative conditions). Thirty-four patients were excluded from the analysis despite fulfilling the eligibility criteria; however, none were excluded after the surgery or allocation process.

### Intervention

The study protocol was approved by the relevant Bioethical Review Board. All methods used in this study were performed in accordance with approval from the Bioethical Committee of the Centre of Postgraduate Medical Education in Warsaw (approval number 13/PB/2016). Written informed consent was obtained from all patients. The patients were prepared for surgery using a typical protocol. LMWH was administered for thromboprophylaxis in doses adjusted for body weight and risk factors. The first heparin dose was administered in the evening of the day before the surgery. Thromboprophylaxis was continued for 35 days after the surgery. All patients received intravenous tranexamic acid (Exacyl) at a dose of 15 mg per kilogram of body weight, 10 min before skin incision. All the patients underwent spinal anesthesia. Experienced surgeons performed the surgeries using a posterolateral approach with cementless hip replacement implantation.

### Outcomes

The primary outcomes measured were the size of the hip hematoma, hemoglobin level, CRP level, VAS, hip range of movement, wound exudation, soft tissue hematoma, intraoperative bleeding, and blood transfusion. The secondary outcomes were infection, deep vein thrombosis, and readmission at 30 days after surgery. All data were collected by the three main investigators.

The volume of blood lost, along with occult bleeding, was calculated using the Gross formula:

$$ EBVx\left({Ht}_{(0)}-\raisebox{1ex}{${Ht}_{(1)}$}\!\left/ \!\raisebox{-1ex}{${Ht}_{(av)}$}\right.\right), $$

where *EBV* represents the estimated patient’s blood volume, *Ht*_(0)_ represents the preoperative hematocrit, *Ht*_(1)_ represents the hematocrit recorded 24 h after surgery, and *Ht*_*av*_ represents the estimated pre- and postoperative hematocrit values. Despite limitations such as intraoperative fluid transfusion or renal insufficiency, the Gross formula is thought to credibly estimate intraoperative blood loss [10]. Blood loss was assessed and calculated on postoperative day 1 by an unblinded clinician.

Over the first 72 h after surgery, all patients underwent ultrasound scans of their hip joints and the postsurgical wound using an aseptic technique with a linear 3–9 MHz ultrasound transducer. The scans were performed with the patients in the supine position. The ultrasound scan assessed the fluid level at the level of the endoprosthesis neck in its long and transverse axes and detected fluid accumulation in the soft tissues around the incision area in its long and transverse axes. The exudate in the dressing was also noted on postoperative day 3. All examinations were performed by a single clinician.

All laboratory tests of CRP and hemoglobin levels were completed on postoperative day 3. Before surgery and within 72 h after surgery, the range of hip motion was assessed by an experienced clinician for flexion, abduction, adduction, and flexion contracture, and deviations in degrees were noted with a measurement accuracy of 5°. Pain levels were assessed using the VAS in all patients on postoperative day 3, with scores ranging from 0 to 10 points. The patients were discharged depending on their general condition and progress in rehabilitation, usually between postoperative days 3 to 10. Data regarding blood transfusions were collected at discharge. The follow-up period was 30 days, and all patients reached their final follow-up. Data regarding infections, deep vein thrombosis, and readmission were collected at the end of the follow-up period. We performed an intention-to-treat analysis.

### Data analysis

Quantitative data were examined using Student’s *t*-test, and their distributions were assessed using the Shapiro–Wilk test. Qualitative characteristics were assessed via contingency analysis using the chi-squared test with Yates’s correction or Fisher’s *F*-tests, as appropriate. The statistical analyses were performed using SAS version 9.4 (SAS Institute Inc., Cary, NC, USA). Analysis items with *p* < 0.05 were considered statistically significant.

## Results

At the final follow-up, we assessed 100 patients: 50 in the drainage group and 50 in the no-drainage group. The statistical analysis did not reveal any statistically significant differences between the groups in terms of age, sex, body weight, degenerative disease severity on the Kellgren–Lawrence scale, and blood clotting factors (activated partial thromboplastin time [APTT], prothrombin time, and international normalized ratio) (Table 1).

**Table 1:** Distribution of basic parameters in both groups

	Drainage mean (range)	No-drainage mean (range)	*p*-value
Age, years	63.1 (39–80)	62.5 (30–80)	0.75
Sex (M/F)	24/26	21/29	0.69
Body weight, kg	83.5 (52–124)	81.7 (56–110)	0.54
APTT, s	26.5 (16.1–37.2)	26.4 (18.1–40.8)	0.93
PT, s	12.4 (8.8–15.1)	12.5 (9.1–15.1)	0.84
INR	1 (0.8–1.3)	1 (0.8–1.3)	0.89

*APTT* activated partial thromboplastin time, *INR* international normalized ratio, *M/F* male/female, *PT* prothrombin time

The investigated patients included 55 women (55 %) and 45 men (45 %). The mean age of the patients was 62.8 years (range, 30–82), 64 years among women (range, 34–76), and 61.4 years among men (range, 30–82). The mean body mass index was 29 kg/m^2^ (29.3 and 28.7 kg/m^2^ in men and women, respectively).

### Primary outcomes

The mean thickness of the fluid assessed at the level of the endoprosthesis neck in the drainage group was 10.3 mm while that in the no-drainage group was 9.8 mm (mean difference, 0.6 mm; 95 % confidence interval [CI] -2.9 to 3.8; *p* = 0.653).

The presence of hematomas in postoperative wound soft tissues, both suprafascial and subfascial, was assessed. Hematomas were detected in 7 patients in the no-drainage group and 8 patients in the drainage group (*p* = 0.78).

In both groups, hemoglobin and CRP values were measured preoperatively and 24 and 72 h postoperatively. At 72 h after surgery, there were no differences in hemoglobin values: 10.9 vs. 11.1 g/dl for the drainage and no-drainage groups, respectively (mean difference, 0.2 g/dl; 95 % CI -2.1 to 2.5; *p* = 0.53). The mean decreases in hemoglobin values before surgery and 72 h postoperatively between groups were not significant (0.8 vs. 0.8 in the no-drainage and drainage groups, respectively (*p* = 0.49). The CRP values measured 72 h postoperatively were also analyzed: 147.6 vs. 131 mg/l for the drainage and no-drainage groups, respectively (mean difference, 16.6 mg/l; 95 % CI -11.3 to 44.5). There were no statistically significant differences between the groups (*p* = 0.33).

The overall mean perioperative blood loss volume was 1175 ml (range, 371–2384). The mean volume of blood loss was 1126 ml in the no-drainage group and 1224 ml in the drainage group (mean difference, 97.1 ml; 95 % CI -84.1 to 278.2; *p* = 0.59). Overall, blood transfusion was rarely needed, with a median of zero cases. In each group, blood transfusion was necessary for five patients (*p* = 0.247).

The clinical status was assessed using the VAS to evaluate pain before surgery and 72 h after surgery. The median VAS scores before surgery were 6 and 7 points in the no-drainage and drainage groups, respectively (mean difference, 0.6; 95 % CI -2 to 3.2). After surgery, the median VAS scores were 5 and 6 points in the no-drainage and drainage groups, respectively (mean difference, 0.4; 95 % CI -2.3 to 3.1; *p* = 0.71) (Table 2).

**Table 2:** Hip range of movement in both groups

	Drainage mean (±SD)	No-drainage mean (±SD)	*p*-value	Mean difference (95 % CI)
Flexion before surgery	85.8 (17.7)	84 (18.2)	0.61	1.8 (-5.4 to 9)
Flexion on POD 3	74.9 (12.9)	78.5 (13.3)	0.5	3.6 (-1.6 to 8.8)
Abduction before surgery	20.7 (9.3)	18.4 (12.2)	0.23	2.3 (-1.8 to 6.4)
Abduction on POD 3	19.8 (7.9)	19.3 (8.3)	0.54	0.5 (-2.8 to 3.8)
Adduction before surgery	9.7 (8.4)	9.1 (8)	0.61	0.6 (-2.7 to 3.9)
Adduction on POD 3	7.1 (8.2)	7.8 (7.6)	0.51	0.7 (-2.5 to 3.9)
Flexion contracture before surgery	6.9 (10.5)	7.3 (10.4)	0.82	0.4 (-3.8 to 4.6)
Flexion contracture on POD 3	5 (9.4)	3.6 (8.6)	0.28	1.4 (-2.2 to 5)

*CI* confidence interval, *POD* postoperative day, *SD* standard deviation

The second clinical indicator evaluated was the hip range of motion 72 h after surgery. Due to varying severities of degenerative disease, we considered differences in the range of motion before surgery and 72 h after surgery. There were no significant differences in any of the movements tested between groups.

### Secondary outcomes

There were no significant differences between the groups in terms of wound exudation on postoperative day 3 (*p* = 0.62). In the no-drainage group, early infection was observed in two patients. Infection was detected as a result of prolonged wound leakage (over 5 days) and was confirmed intraoperatively with two positive bacteriological cultures. These patients received the debridement, antibiotics, and implant retention (DAIR) procedure with good outcomes. There were no significant differences between the groups in the incidence of infection (*p* = 0.47).

The risk of deep vein thrombosis within 30 days after hip replacement surgery was also assessed. None of the patients in either group experienced postoperative deep vein thrombosis.

The mean hospitalization duration in both groups was 7 days; the mean difference between groups was 0.3 days (95 % CI -2.3 to 2.9; *p* = 0.60). Because of prolonged wound leakage, 2 patients were readmitted within the 30-day period after surgery and qualified for the DAIR procedure. These patients were diagnosed with early infection (*p* = 0.49) (Table 3).

**Table 3:** Primary and secondary outcomes in both groups

	Drainage	No-drainage	*p*-value	Mean difference (95 % CI)
Hip hematoma size in USG Mean (±SD) (mm)	10.3 (7.8)	9.8 (9)	0.653	0.5 (-2.8 to 3.9)
Hb level on POD 3 Mean (±SD) (mg/dl)	10.9 (5.7)	11.1 (5.6)	0.53	0.2 (-2.1 to 2.5)
CRP level on POD 3 Mean (±SD) (mg/l)	131 (63.6)	147.6 (75.2)	0.33	16.6 (-11.3 to 44.5)
VAS on POD 3 Mean (±SD)	4.9 (2.2)	5.3 (2.3)	0.71	0.4 (-2.3 to 3.1)
Wound exudation on POD 3 (no.)	9	11	0.62	-
Soft tissue hematoma on POD 3 (no.)	8	7	0.78	-
Intraoperative bleeding Mean (±SD) (ml)	1224 (438.2)	1126 (466.2)	0.59	98 (-84.1 to 278.2)
Blood transfusion (no.)	5	5	0.247	-
Infection (no.)	0	2	0.47	-
Deep vein thrombosis (no.)	0	0	-	-
Readmission within 30 days (no.)	0	2	0.49	-

*CI* confidence interval, *CRP* C-reactive protein, *Hb* hemoglobin, *POD* postoperative, *SD* standard deviation, *USG* ultrasonography, *VAS* Visual Analog Scale

## Discussion

We assessed a homogenous group of patients who underwent hip replacement surgery. For legal reasons, all patients received extended thromboprophylaxis. The safety of suction drainage has not been evaluated in this group of patients. The parameter that might provide information about the impact of preoperative heparin dose was the amount of blood in the hip joint postoperatively. In our analysis, we checked fluid levels above the endoprosthesis neck using ultrasonography in the supine position. We found no differences in hip fluid levels between those who received suction drainage and those who did not (9.8 vs. 10.3 mm, *p* = 0.653). In addition, in comparing hematoma formation in soft tissue, intraoperative bleeding including occult bleeding, hemoglobin levels after surgery, and wound healing, we found no differences between the groups. Based on this, we concluded that refraining from placing suction drainage has no effect on patients receiving extended thromboembolic prophylaxis who undergo primary cementless hip replacement.

Due to the short observation period, the VAS and range of hip motion were used to assess patient-related outcomes. The VAS is commonly used to assess pain experienced by patients undergoing hip replacement surgery [11]. In a meta-analysis, Hou et al. did not demonstrate any differences in VAS scores between groups [12]. Fagotti et al. found significantly higher VAS scores among patients without suction drainage than among those with drainage [9]. In our study, 72 h postoperatively, a non-significant difference between the groups was identified in the VAS scores. Hip joint hematoma can reduce the range of motion [13]. Zeng et al. demonstrated a reduced range of hip joint mobility following surgery in patients among whom suction drainage was not placed [14]. Nevertheless, in a meta-analysis, Chen et al. did not find any differences in the range of motion between the drainage and no-drainage groups, as was confirmed in our study [15].

In the no-drainage group, we observed two patients with deep infections, whereas, in the drainage group, there were no infections. No surgical site infections (SSIs) were observed. Only two patients experienced prolonged wound leakage (> 5 days); however, in these cases, we recognized deep infections. We also did not find any significant differences in CRP levels. Historically, suction drainage was justified by the need to reduce hip joint hematoma and was consequently used to reduce the risk of periprosthetic infection [16]. The first papers to question the use of suction drainage were published in the 1990 s [17, 18]. Hou et al., based on 27 randomized studies, did not demonstrate a higher incidence of infections in patients without drainage [12]. Similar conclusions were drawn by Chen et al. based on 16 papers [15]. Although these studies showed findings similar to ours, Fagotti et al. reported two SSIs in the drainage group but no deep periprosthetic infections; in the no-drainage group, there were no SSIs, but one patient was diagnosed with deep infection. However, there were no significant differences between the groups [9]. In a study of 552 patients (577 hip joints), Walmsley et al. reported a higher incidence of SSIs (48 % vs. 2.9 %) and deep infections (0.7 % vs. 0.4 %) in the no-drainage group [19]. Zimmerli et al. questioned the diagnosis of SSI in patients with implants because it cannot be clinically differentiated from deep infections [20]. Despite the absence of statistically significant differences, this raised doubts about the non-use of suction drainage in patients undergoing extended thromboprophylaxis, which needs further evaluation.

We did not find an increased need for blood transfusions in either group. However, in a meta-analysis, Kelly et al. demonstrated that patients in the suction drainage group required blood transfusion significantly more frequently and had greater postoperative blood loss [21]. In another meta-analysis, Hou et al. also demonstrated significantly more frequent blood transfusions in the drainage group [12].

In most relevant publications, the majority of parameters did not differ significantly between groups [9, 19, 21, 22]. All these studies included heterogeneous groups: patients with primary and secondary osteoarthritis, coxarthrosis in the course of rheumatoid arthritis, and others. Some of these conditions can affect perioperative blood loss and the need for blood transfusion [23]. Despite this, because of Polish recommendations, some groups of patients undergoing extended thromboprophylaxis require longer heparin use due to risk factors for thrombosis. These patients could have achieved some benefits with the use of suction drainage. These conditions illustrate why further well-designed multicenter prospective studies are needed.

This study had several limitations. First, it was a single-center study and therefore may be subject to selection bias. For this reason, we instituted strict inclusion and exclusion criteria. Therefore, multicenter studies are needed to validate our findings. A short observation period was appropriate for the intervention investigated. Therefore, we used proper scales to assess the short-term clinical outcomes. Many patients were excluded from the primary cohort, possibly influencing outcomes; however, all exclusions occurred before we opened the sealed envelopes, which ultimately did not significantly impact the randomization process. All therapists and assessors were blinded; however, at the point of ultrasonography examination and clinical evaluation (hip range of motion, VAS) on postoperative day 3, the use of suction drainage was visible, which could potentially have affected their assessment. Another limitation was the retrospective registration at ClinicalTrials.gov.

## Conclusions

Hip replacement without suction drainage after surgery is a recognized therapeutic method. No superiority of either method was demonstrated in terms of the size of the hip hematoma, hemoglobin levels, CRP levels, VAS, hip range of movement, wound exudation, soft tissue hematoma, intraoperative bleeding, and blood transfusion. In light of these results, we recommend not routinely using suction drainage in patients undergoing extended thromboprophylaxis. Nevertheless, it is worth noting that there were two cases of early infection in the no-drainage group compared to none in the drainage group among patients who underwent hip replacement with extended thromboprophylaxis. This finding suggests the need for further research.

## Data Availability

The datasets used and/or analyzed during the current study are available from the corresponding author upon reasonable request.

## Abbreviations

APTT: Activated partial thromboplastin time
CI: Confidence interval
CRP: C-reactive protein
DAIR: Debridement, Antibiotics, Implant Retention procedure
LMWH: Low-molecular-weight heparin
SSIs: Surgical site infections
VAS: Visual analog scale
